# Supplementary material for: Holocene sea ice variability driven by wind and polynya efficiency in the Ross Sea
Source: Nat Commun. 2017 Nov 6;8:1334. doi: 10.1038/s41467-017-01455-x (PMC5673891; doi:10.1038/s41467-017-01455-x)
Supplement: Supplementary file 1 — Supplementary Information [file 41467_2017_1455_MOESM1_ESM.pdf]

Supplementary Information

Supplementary Note 1: marine core chronology

Supplementary Table 1. Table with the radiocarbon dated material. Conventional and calibrated ^{14}C ages analysed on the acid-insoluble organic matter (AIOM) and one mollusc shell for the studied cores and box-cores.

Core and box-core	Location	Sample depth (cm)	Sediment facies[a]	Carbon source [b]	Laboratory code	Conventional ^{14}C age (yr BP)	∂^{13}C	LCO Age (yr) [c]	Calibrated age (yr BP) [d]	1 σ range	
										Min	Max
ANTA99-cJ5	Joides Basin	5-6	DO	AIOM	From ref 1	5000 ±30	-30.6	2637±131	**1121**	931	1289
ANTA99-cJ5	Joides Basin	59-60	DO	AIOM	From ref 1	6120±40	-30.6	2637±131	**2406**	2205	2665
ANTA99-cJ5	Joides Basin	139-140	DO	AIOM	From ref 1	8620±40	-29.5	2637±131	**5545**	5317	5733
ANTA99-cJ5	Joides Basin	240-241	TGM	AIOM	From ref 1	13,850±50	-	2637±131	**11,597**	11,242	11,856
BAY05-bc21	Cape Hallett	0-1	DO	AIOM	OS-90305	1580±45	-25.29				
BAY05-20c	Cape Hallett	1-2	DO	AIOM	OS-59367	1760±30	-25.4	436±129	**203**	0	275
BAY05-20c	Cape Hallett	146-147	DO	AIOM	OS-59368	2760±40	-26.0	436±129	**1083**	911	1267
BAY05-20c	Cape Hallett	240-242	DO	mollusc	OS-59019	2640±30	0.4		**1411**	1278	1539
BAY05-20c	Cape Hallett	368-369	DO	AIOM	OS-59369	4750±30	-26.0	436±129	**3408**	3178	3630
BAY05-bc40	Wood Bay	0-1	DO	AIOM	OS-90283	2230±30	-24.24				
BAY05-43c	Wood Bay	100-101	DO	AIOM	OS-90285	2580±30	-24.42	1086±125	**327**	186	494
BAY05-43c	Wood Bay	200-201	DO	AIOM	OS-90286	2960±25	-24.84	1086±125	**665**	503	808
BAY05-43c	Wood Bay	300-301	DO	AIOM	OS-90287	3550±30	-25.48	1086±125	**1223**	1029	1401
BAY05-43c	Wood Bay	376-377	DO	AIOM	OS-96502	5710±35	-24.73	1086±125	**3798**	3561	4020
BAY05-43c	Wood Bay	407-408	TGM	AIOM	OS-90288	7200±40	-25.02	1086±125	**5697**	5511	5896

a. DO = diatomaceous ooze; TGM = transitional glaciomarine sediment; **b.** AIOM = acid-insoluble organic matter; **c.** LCO = local contamination offset (see text); **d.** calibrated ages using CALIB REV 7.1.0 and the MARINE 13 calibration curve[2] applying modern regional effect (MRE) of 1144±121 years with a ΔR of 791±121 years[3]. All the ages are in years BP (1950 AD).

Chronology of carbonate barren sediments

The marine cores' chronology development followed the methodology detailed in the literature. The apparent ages of modern sediments in Western Ross Sea combine the regional marine reservoir effect (MRE) and the local dead carbon (LCO) contamination. The protocol that is generally applied

in these environments is to remove the modern mean reservoir effect to quantify the local DC fraction. Both the MRE and DC contamination are generally propagated down-core.

We below present arguments that support the marine cores' chronology along with a sensitivity test demonstrating that the paleoceanographic interpretation is still valid at the dating and MRE and DC uncertainties.

Mean reservoir age and dead carbon contamination

We used box cores located at the same location of WRS_CH and WRS_WB marine cores to calculate the DC contamination. The box cores preserved the interface (~0 BP) as their on-board description showed the presence of the fluffy layer (Supplementary Fig. 1). No twin box core was available at site WRS_JB. We thereafter explain how we cope with this deficiency.

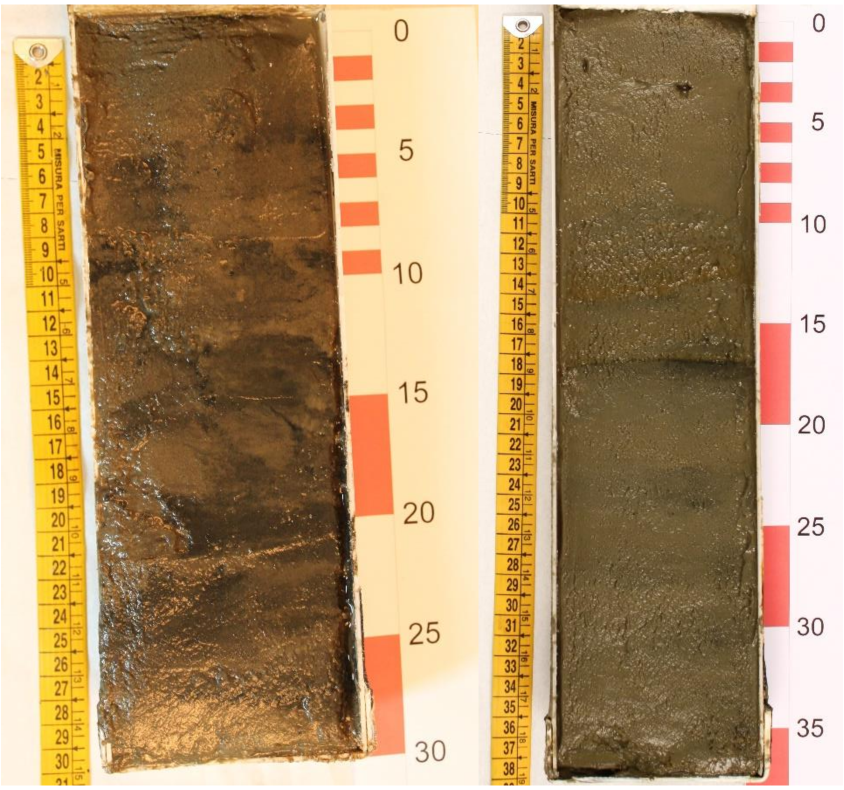

Supplementary Figure 1. Photos of box core BAY05-bc21 (left) and BAY05-bc40 (right) showing few millimetres of oxidized sediments at the core-top which represent the most recent sedimentation.

Mean reservoir age

We here applied the mean reservoir age calculated by Ref. 3, from the difference between U/Th and ^{14}C in corals in Southwestern Ross Sea, a modern reservoir age of 1000-1300 years with a mean value of 1144±121 years. This value is very close to previous data obtained from pre-bomb historical samples[4] (1131±125 years). These measures are performed on coral samples and are DC free. It is important to note that no changes in the mean difference and standard deviation between U/Th ages and ^{14}C ages have been observed during the past 6000 years. Similarly, Ref. 5 found a constant ~400 years difference between calcite ^{14}C dates and acid-insoluble organic matter ^{14}C dates off Adélie Land over the last 4000 years. The application of a constant reservoir age over the mid- to late-Holocene used in our study is therefore reasonable. This MRE is applicable to the three sites that derive their surface waters from the same main water masses.

Dead Carbon WRS_CH and WRS_WB

Using the raw ^{14}C dates in surface sediments of box cores at the coastal core sites and the regional MRE (1144±121 years), we calculated DC pools of 436 years at Cape Hallett (WRS_CH) and 1086 years at Wood Bay (WRS_WB) with a 1σ error of ±129 and ±125 years, respectively. They appear younger than the ~2000 years difference between fatty acids and AIOM ages in Southwestern Ross Sea[6]. We think that this is due to the distance from the main DC source represented by the Ross Ice Shelf (RIS)[7]. Indeed, the three cores here investigated are north of the area studied by Ref. 6 and 7. In the Ross Sea, the DC decreases from South to North and from East to West. Cape Hallett is here the northernmost site (650 km from RIS) inside a bay, having a South-North orientation, where the RIS supplied DC is very low. To the South, we have Wood Bay site (WRS_WB) located 400 km away from RIS in an environment similar to WRS_CH site. WRS_JB core is located in Joides Basin (in the middle of the Ross Sea at around 400 km from RIS). The regional pattern in DC contamination

observed here is supported by micropalaeontological data. Indeed, the proportion of extinct diatoms (a proxy of a DC contribution from RIS) is very low at WRS_JB (<2%) and negligible at WRS_CH and WRS_WB. Moreover, the continental DC on the coastal bays may be diluted by the local high productivity (see Figure 7 in the main text).

Dead Carbon WRS_JB

Unfortunately, no box core was retrieved at WRS_JB core site. However, a set of nearby trigger cores (distance less than 100 km), in which the core-tops were preserved have been sampled in the Joides Basin[9]. The AIOM ^{14}C dates in these trigger core-tops[9] show a range between 3339 and 4439 years BP (Sample ID: 94T16, 94T17, 94T18, 94T20, 95K39) resulting in a mean age of 3781±51 years BP with a standard deviation of ±608 years. From the mean core-top age (3781±51 yr BP) and MRE (1144±121 yr) we calculated a mean LCO value of 2637±131 years (1 σ error).

Constant age offset

The lithology of our cores provides additional support to the constant age offset between AIOM and calcite dates evidenced in published studies from East Antarctica[5,9]. Indeed, reasonably homogeneous lithology and C_{org} content, C/N ratio, as well as a very low content (maximum value of 0.28% for coastal sites WRS_WB/WRS-CH and 1.43% for WRS_JB) of reworked diatoms (see Figure 7 in the main text) characterise each core, arguing against drastic changes in amount and origin of the terrigenous supply and, thus, against drastic changes in DC supply to the core sites. Additionally, one ^{14}C date on a mollusc shell at 241 cm in WRS_CH core gives the possibility to cross-check our AIOM dates. The difference between the age obtained at 147 cm (0.86 ka) by the interpolation between the mollusc age and the surface and the AIOM ages (1.08 ka) is around 200 years.

Chronology uncertainties marine vs ice cores

The data support that the uncertainties in marine chronologies are at maximum 600 years. We performed a sensitivity test in which we allowed the geological and ice core records to move temporally (200, 400 and 600 years) along their respective chronological uncertainties (Supplementary Fig. 2). This sensitivity test demonstrates the correlation between *F. curta* coastal marine cores (WRS_WB, WRS-CH) and TY ssNa do not have large variation and remain stable inside ±600 years and that the main interpretations, here dealing with pluri-centennial climate variations, are not altered.

The *F. curta* records are compared to the ice core records on their mean chronology along with the "error" (horizontal bars): 600 years for marine cores and 100 years maximum for ice cores over the Holocene. The dating accuracy for the ice cores is ±1 year in coincidence with historical volcanic eruptions (last 2000 yr) up to 100 years in points that are far from dated reference horizons. The anti-correlation between the coastal and open ocean cores will still persist even if cores are moved in opposite temporal direction by the maximum "error" (Supplementary Fig. 2). Similarly, the *F. curta* records in coastal cores still compare well to Taylor Dome ssNa record while the *F. curta* record in the open ocean core still compares well to TALDICE ssNa record.

It is worth noting that the only way to reconcile the three marine records is to make the coastal cores older by 5000 years. In this way, the *F. curta* increasing trends in WRS_CH and WRS_WB would span the 9000-5000 years BP period, aligning with the WRS_JB core. This is impossible for the reasons explained above and for the close resemblance between the *F. curta* records in WRS_CH and WRS_WB with *F. curta* records in other coastal cores from East Antarctica[10,11,12]. Additionally, further support comes from the environmental reconstructions inferred from diatom counts that agree well with independent macrofaunal reconstructions[13,14] and deglaciation history of WRS[15].

Many papers in the literature tune open Southern Ocean sea-surface temperature records to ice core $\delta^{18}O$ or δD records (see Ref. 16 for example) or open Southern Ocean Fe records to ice core dust concentration records (see Ref. 17 for example) inferring a concomitant response of the Southern Ocean and Antarctica over the past climatic cycles. Without any adjustment, we here show that such synergy did exist for the Holocene. As such, taking into account the maximum inaccuracy of the marine and ice core records does not change our main interpretations because we do not focus on rapid climate change but on multi-centennial to millennial trends.

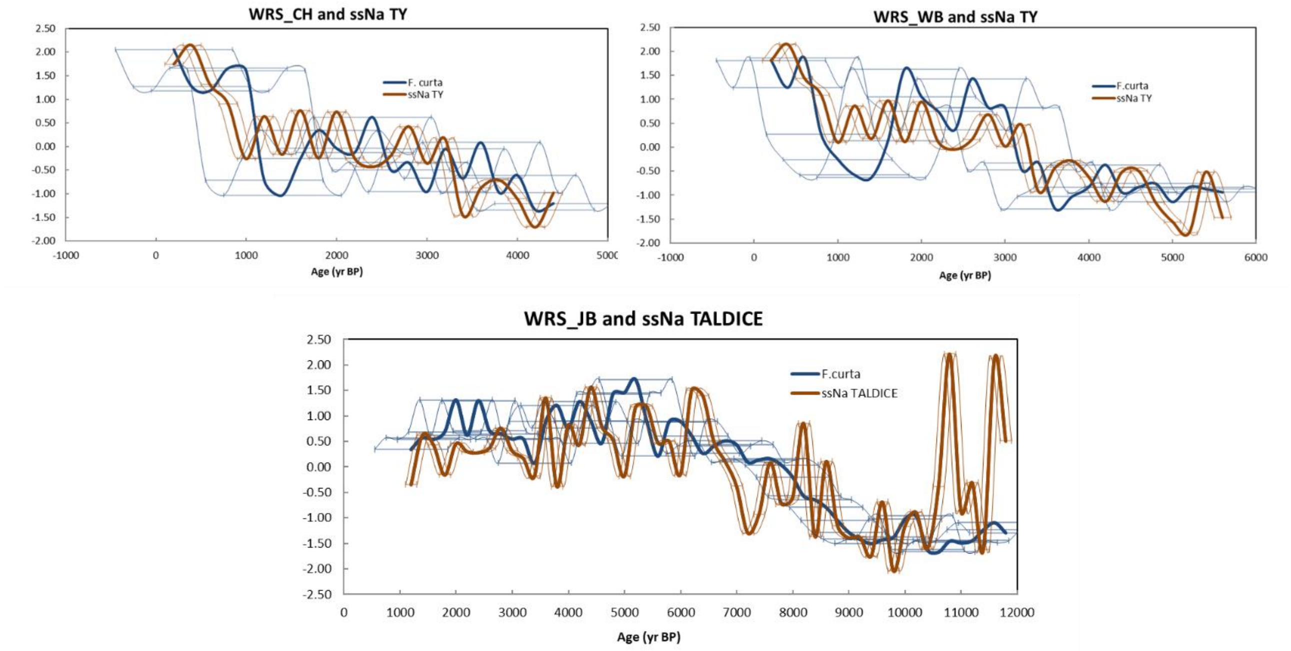

Supplementary Figure 2. Comparison between WRS_CH and WRS_WB *F. curta* relative abundances to ssNa TY (upper panels) and comparison of WRS_JB *F. curta* relative abundances to ssNa TALDICE (bottom panel). All data are resampled on a 200-yr time step and standardized for the considered periods. The horizontal error bars of ±600 years are used for the marine core data, while bars of ±100 years are used for the ice core data.

Benefit and flaws of new dating techniques

The most common new dating techniques that could have been applied to carbonate barren sediments are ^{14}C of fatty acids[6,7,8,18] or programmed-temperature pyrolysis[19]. Both techniques provide more robust dates by alleviating most of the DC issues, but we cannot perform further analyses. It is worth noting though that ^{14}C of fatty acids can also be altered by old carbon as dates

as old as 22 ka have been measured in the laminated sections above the diamicton at site U1357A off Adélie Land (Ohkouchi, personal communication).

Supplementary Note 2: ^{14}C dated faunal remains (Adélie penguins, elephant seals and silverfish)

More than 1/3 of the world Adélie penguin population (*Pygoscelis adeliae*) settles in the Western Ross Sea along the Victoria Land Coast (VLC), occupying one of the southernmost ecosystems on the Earth[20].

Penguin guano is seeped through the permeable pebbly nests on surficial deposits and accumulates to form thick guano layers. In long-term occupied penguin colonies, the accumulation of droppings, feathers, egg fragments, and bird carcasses produces ornithogenic soils[21].

The identification of several abandoned nesting sites in the outskirts of presently occupied colonies testifies the endurance of favourable conditions for penguin life and settlement and variations in penguin population through time. Furthermore, abandoned penguin nesting sites found in areas where the Adélies do not nest at present along the VLC have been recognized as relict colonies[22].

Relict colonies and abandoned nesting sites near presently occupied colonies are among the richest resources of organic matter in coastal areas. Organic remains are well preserved in the cold and dry Antarctic environment and retain precious information for reconstructing penguin occupation of the Antarctic coasts. They also supplied relevant radiocarbon dates (more than 450 from VLC areas; Supplementary Fig. 3) providing an Adélie penguin history spanning the last 8 ka[22-38].

The excellent preservation of prey items remains in ornithogenic soils can also give considerable information on Adélie penguin paleodiet, on their foraging strategies and, consequently, on the paleoecology and paleoclimatology of the Antarctic ecosystem. In fact, due to ecological competition, variation of fish exploitation respect to krill in Adélie penguin diet would reflect sea ice extent and persistence[14,39].

Skin and mummified remains, also identified with DNA analysis, reveal that southern elephant seals (*Mirounga leonina*) once inhabited the VLC of the Ross Sea[13,40,41]. Today, elephant seals live in the sub-Antarctic and the closest extant rookery lies 3400 km to the VLC on Macquarie Island.

More than 260 radiocarbon dates have been obtained from abandoned southern elephant seal colonies along the VLC[13,23,24,40,42,43].

The presence of southern elephant seal colonies is interpreted at least to have been induced by significant decrease in coastal sea ice if not by the penetration of sub-Antarctic-like conditions into the Ross Sea embayment[24] (Supplementary Fig. 3).

All the radiocarbon dates are calibrated with Calib Rev 7.0[2] applying a ΔR of 0.79±0.12 ka[3].

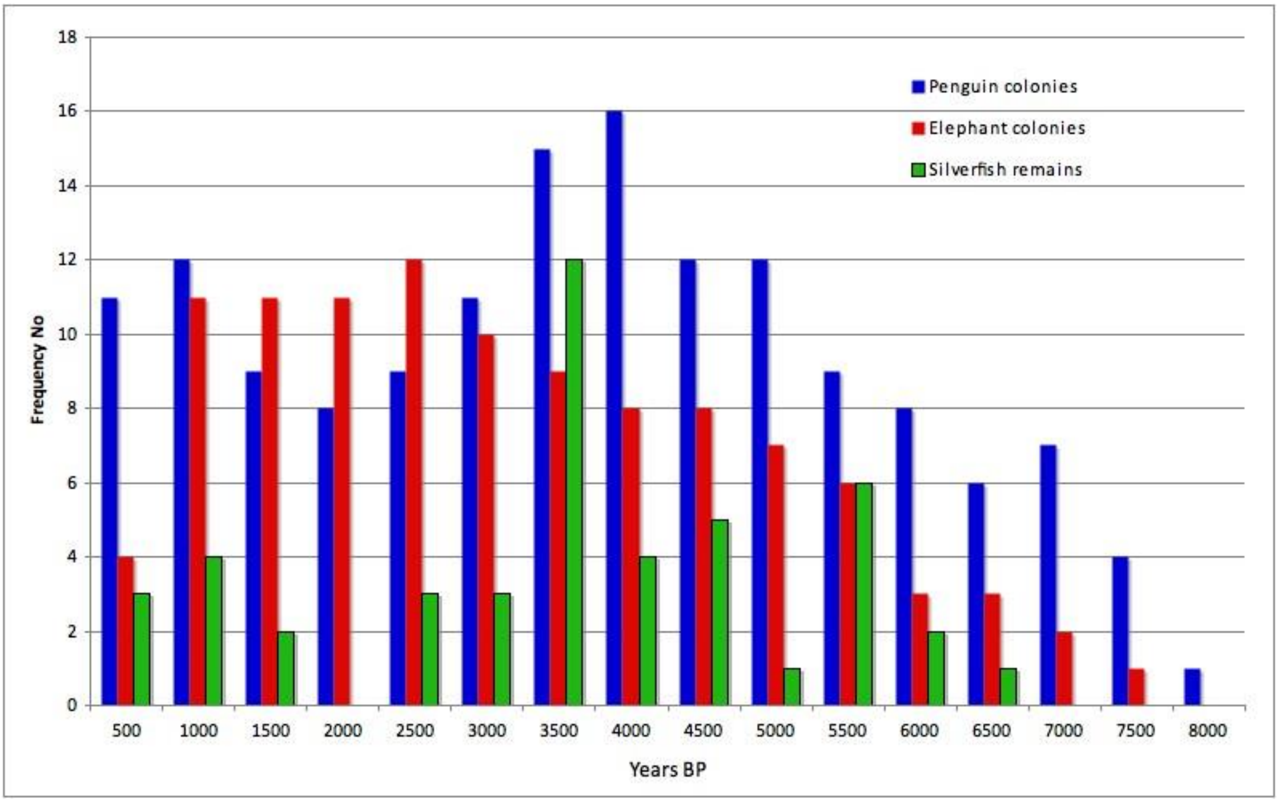

Supplementary Figure 3. Frequency and distribution of i) Adélie penguin colonies (both presently occupied and relict colonies; blue bars); ii) elephant seals colonies (red bars); iii) fish remains (mainly *Pleurogramma antarcticum*; green

bars) from guano layers collected in abandoned penguin nesting sites and relict colonies. Frequency of penguin colonies, abandoned elephant seal colonies, and samples with fish remains are grouped each 0.5 ka.

Supplementary Note 3: Ice core data

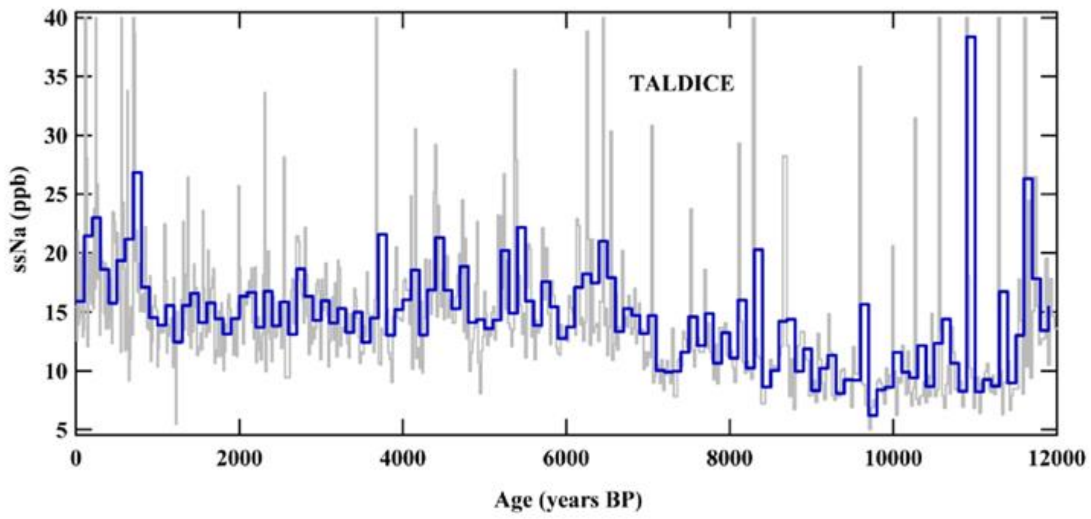

Supplementary Figure 4. ssNa contents (raw data in grey, in ppb, part per billion; this study) for TALDICE ice core along with resampled data at 100-year step (dark blue line).

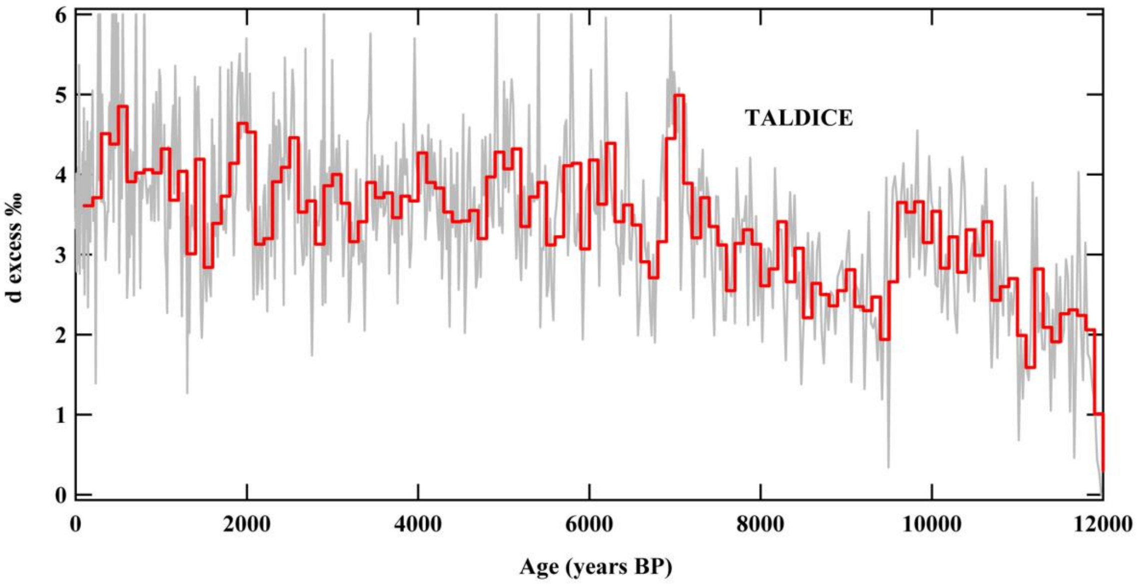

Supplementary Figure 5. Deuterium excess (raw data in grey, this study) for TALDICE ice core along with resampled data at 100-year step (red line).

Variability of deuterium excess and $\delta^{18}O$ at TALDICE and Taylor Dome

The variability of the isotopic data was calculated over two consecutive periods, 0-7 and 7-12 ka and reported in the table below. The raw data were resampled at 50 years and detrended.

Supplementary Table 2. Standard deviation values in unit per mil (‰) for TALDICE and Taylor Dome isotopic data

**TALDICE**	**0-7 ka**	**7-12 ka**
**$\delta^{18}O$**	0.76	0.61
**d exc**	0.96	0.88
**Taylor**		
**$\delta^{18}O$**	0.93	0.60
**d exc**	1.27	0.71

The variance of Taylor Dome deuterium excess is significantly larger during 0-7 ka compared to 7-12 ka and larger than that at TALDICE.

Supplementary Note 4: Correlation analysis tables

Supplementary Table 3. Correlation coefficients (r) obtained between the diatom *F. curta* from the marine core WRS_JB (Joides Basin) and ice core proxies. All the data were resampled at a common 200-year step and standardized for the period from 1.2 to 11.8 ka. The style used to report the correlation coefficients (r) depends on the p-value: standard style for p<0.05, *italics* for 0.05<p<0.02 and **bold** for p<0.01.

	F. curta WRS_JB
F. curta WRS_JB	1
ssNa TALDICE	**0.49**
ssNa TY	**-0.78**
d TALDICE	**0.77**
d TY	**0.57**
$\delta^{18}O$ TALDICE	**0.41**
$\delta^{18}O$ TY	**-0.66**

Supplementary Table 4. Correlation coefficients (r) obtained between the diatom *F. curta* from the marine core WRS_CH (Cape Hallett) and ice core proxies. All the data were resampled at a common 200-year step and standardized for the period from 0.2 to 4.4 ka. The style used to report the correlation coefficients (r) depends on the p-value: standard style for p<0.05, *italics* for 0.05<p<0.02 and **bold** for p<0.01.

	F. curta WRS_CH
F. curta WRS_CH	1
ssNa TALDICE	**0.56**
ssNa TY	**0.67**
d TALDICE	*0.50*
d TY	-0.11
δ^{18}O TALDICE	-0.30
δ^{18}O TY	*-0.47*

Supplementary Table 5. Correlation coefficients (r) obtained between the diatom *F. curta* from the marine core WRS_WB (Wood Bay) and ice core proxies. All the data were resampled at a common 200-year step and standardized for the period from 0.2 to 5.6 ka. The style used to report the correlation coefficients (r) depends on the p-value: standard style for p<0.05, *italics* for 0.05<p<0.02 and **bold** for p<0.01.

	F. curta WRS_WB
F. curta WRS_WB	1
ssNa TALDICE	0.28
ssNa TY	**0.71**
d TALDICE	*0.38*
d TY	0.26
δ^{18}O TALDICE	0.25
δ^{18}O TY	**-0.57**

Supplementary Table 6

Correlation coefficients (r) obtained between the diatom *F. curta* from the marine cores WRS_JB (Joides Basin), WRS_WB (Wood Bay) and WRS_CH (Cape Hallett). All the data were resampled at a common 200-year step and standardized for the period from 1.2 to 5.6 ka for WRS_JB- WRS_WB, from 1.2 to 4.4 ka for WRS_JB- WRS_CH and from 0.2 to 4.4 ka for WRS_CH- WRS_WB. The style used to report the correlation coefficients (r) depends on the p-value: standard style for p<0.05, *italics* for 0.05<p<0.02 and **bold** for p<0.01.

	F. curta WRS_JB	*F. curta* WRS_CH	*F. curta* WRS_WB
F. curta WRS_JB	1		
F. curta WRS_CH	0.10	1	
F. curta WRS_WB	0.14	**0.52**	1

Supplementary Table 7. Correlation table considering ice core records. All the data were resampled at a common 200-year step and standardized for the considered period 1.0 to 11.6 ka. The style used to report the correlation coefficients (r) depends on the p-value: standard style for p<0.05, *italics* for 0.05<p<0.02 and **bold** for p<0.01.

	ssNa TALDICE	ssNa TY	d TALDICE	d TY	δ^{18}O TALDICE	δ^{18}O TY
ssNa TALDICE	1					
ssNa TY	**-0.36**	1				
d TALDICE	*0.25*	**-0.72**	1			
d TY	*0.25*	**-0.58**	**0.55**	1		
δ^{18}O TALDICE	0.22	*-0.31*	**0.38**	**0.48**	1	
δ^{18}O TY	*-0.27*	**0.38**	**-0.55**	**-0.47**	**-0.59**	1

Supplementary References

[1] Salvi, C., Salvi, G., Stenni, B. & Brambati, A. Paleoproductivity in the Ross Sea during the last 15kyr BP and its link with ice-core temperature proxies. *Ann. Glaciol*. **39**, 1-7 (2004).

[2] Reimer, P. J. *et al.* INTCAL13 and MARINE13 radiocarbon age calibration curves 0–50,000 years CAL BP. *Radiocarbon* **55**, 1869-1887 (2013).

[3] Hall, B.L., Henderson, G. M., Baroni, C. & Kellogg, T.B. Constant Holocene southern-ocean 14C reservoir ages and ice-shelf flow rates. *Earth Planet. Sci. Lett*. **296**, 115-123 (2010).

[4] Berkman, P., Forman, S., 1996. Pre-bomb radiocarbon and the reservoir correction for calcareous marine species in the Southern Ocean. *Geophys. Res. Lett.* **23**, 363–366.

[5] Costa, E. *et al*., Solar forcing and El Niño-Southern Oscillation (ENSO) influences on productivity cycles interpreted from a late Holocene high-resolution marine sediment record, Adélie Drift, East Antarctic Margin. *USGS OF-2007-1047*, Short research Paper 036, doi: 10.3133/of2007-1047-srp036 (2007).

[6] Ohkouchi, N., Eglinton, T. I. & Hayes, J. M. Radiocarbon dating of individual fatty acids as a tool for refining Antarctic margin sediment chronologies. *Radiocarbon* **45**, 17–24 (2003).

[7] Ohkouchi, N. & Eglinton, T. I. Radiocarbon constraint on relict organic carbon contributions to Ross Sea sediments. *Geophys. Geosyst.* **7**, Q04012, doi:10.1029/2005GC001097 (2006).

[8] Ohkouchi, N. & Eglinton, T. I. Compound-specific radiocarbon dating of Ross Sea sediments: a prospect for constructing chronologies in high-latitude oceanic sediments. *Quat. Geochronol.* **3**, 235-43 (2008).

[9] Andrews, J. T. *et al.* Problems and possible solutions concerning radiocarbon dating of surface marine sediments, Ross Sea, Antarctica. *Quat. Sci. Res.* **52**, 206-216 (1999).

[10] Cunningham, W., Leventer, A., Andrews, J. T., Jennings, A. E. & Licht, K. J. Late Pleistocene-Holocene marine conditions in the Ross Sea, Antarctica: evidence from the diatom record. *The Holocene* **9**, 129-139 (1999).

[11] Denis, D. *et al.* Sea ice and wind variability during the Holocene in East Antarctica: insight on middle-high latitude coupling. *Quat. Sci. Rev.* **29**, 3709-3719 (2010).

[12] Campagne, P. Etude de la variabilité des conditions océanographiques et climatiques en Antarctique de l'Est (Terre Adélie – Georges V) au cours de l'Holocène tardif et de la période instrumentale. *PhD thesis*, Université de Bordeaux, pp. 298 (2015).

[13] Hall, B. L. *et al.* Holocene elephant seal distribution implies warmer that present climate in the Ross Sea. *Proc. Natl. Acad. Sci. USA* **103**, 10213-10217 (2006).

[14] Lorenzini, S. *et al.* Adélie penguin dietary remains reveal Holocene environmental changes in the western Ross Sea (Antarctica). *Paleogeogr. Paleoclimatol. Paleoecol.* **395**, 21-28 (2014).

[15] Anderson, J. B. *et al.* Ross Sea paleo-ice sheet drainage and deglacial history during and since the LGM. *Quat. Sci. Rev.* **100**, 31-54 (2014).

[16] Pahnke, K. & Zahn, R. Southern Hemisphere water mass conversion linked with North Atlantic climate variability. *Science* **307**, 1741-1746 (2005).

[17] Martinez-Garcia, A. *et al.* Iron fertilization of the Subantarctic Ocean during the last ice age. *Science* **343**, 1347-1350 (2014).

[18] Yamane, M. et al. Compound-specific 14C dating of IODP expedition 318 core U1357A obtained off the Wilkes Land Coast, Antarctica. *Radiocarbon* **56**, 1-9 (2014).

[19] Rosenheim, B. E. *et al.* Antarctic sediment chronology by programmed-temperature pyrolysis: Methodology and data treatment. *Geochem. Geophys. Geosyst.* **9**, Q04005, doi:10.1029/2007GC001816 (2008).

[20] Ainley, D. G. *et al.* Geographic structure of Adélie penguin populations: overlap in colony-specific foraging areas. *Ecological Monographs* **74**, 159-178 (2004).

[21] Ugolini, F. C. Ornithogenic soils of Antarctica. *Antarct. Res. Ser.* **20**, 181-193 (1972).

[22] Baroni, C. & Orombelli, G. Abandoned penguin rookeries as Holocene paleoclimatic indicators in Antarctica. *Geology* **22**, 23-6 (1994).

[23] Baroni, C. & Orombelli, G. Holocene raised beaches at Terra Nova Bay, Victoria Land, Antarctica. *Quat. Res.* **36**, 157-177. (1991).

[24] Baroni, C. & Hall, B. L. A new Holocene relative sea-level curve for Terra Nova Bay, Victoria Land, Antarctica. *J. Quat. Sci.* **19**, 377-396 (2004).

[25] Dochat, T. M., Marchant, D. R. & Denton, G. H. Glacial geology of Cape Bird Ross Island, Antarctica. *Geogr. Ann. A.* **82**, 237-247 (2000).

[26] Emslie, S. D., Berkman, P. A., Ainley, D. G, Coats, L. & Polito, M. Late-Holocene initiation of ice-free ecosystems in the southern Ross Sea, Antarctica. *Mar. Ecol. Prog. Ser.* **262**, 19-25 (2003).

[27] Emslie, S. D., Coats, L. & Licht, K. A 45,000 yr record of Adélie penguins and climate change in the Ross Sea, Antartica. *Geology* **35**, 61-64 (2007).

[28] Gardner, N., Hall, B. & Wehmiller, J. Pre-Holocene raised beaches at Cape Ross, southern Victoria Land, Antarctica. *Mar. Geol.* **229**, 273-84 (2006).

[29] Hall, B. L. & Denton, G. H. New relative sea-level curves for the southern Scott Coast, Antarctica: Evidence for Holocene deglaciation of the western Ross Sea. *J. Quat. Sci.* **14**, 641-650 (1999).

[30] Hofstee, E. H., Balks, M. R., Petchey, F. & Campbell, D. I. Soils of Seabee Hook, Cape Hallett, northern Victoria Land, Antarctica. *Antarc. Sci.* **18**, 473-486 (2006).

[31] Lambert, D. M. *et al.* Rates of evolution in ancient DNA from Adélie penguins. *Science* **295**, 2270-2273 (2002).

[32] Lorenzini, S. *et al.* Stable isotopes reveal holocene changes in the diet of Adélie penguins in northern Victoria Land (Ross Sea, Antarctica). *Oecologia* **164**, 911-919 (2010).

[33] Lorenzini, S. *et al.* Insights into the Holocene environmental setting of Terra Nova Bay region (Ross Sea, Antarctica) from oxygen isotope geochemistry of Adélie penguin eggshells. *The Holocene* **22**, 63-69 (2012).

[34] Millar, C. D. *et al.* Mutation and evolutionary rates in Adélie penguins from the Antarctic. *PLoS Genet.* **4**, e1000209, doi:10.1371/journal.pgen.1000209 (2008).

[35] Polito, M. J., Emslie, S. D. & Walker, W. A 1000-year record of Adélie penguin diets in the southern Ross Sea. *Antarc. Sci.* **14**, 327-332 (2002).

[36] Parks, M. *et al.* Ancient population genomics and the study of evolution. *Phil. Trans. R. Soc. B* **370**, 20130381 (2015).

[37] Stuiver, M., Denton, G. H., Hughes, T. & Fastook, J. L. History of the Marine Ice Sheet in West Antarctica during the last glaciation: a working hypothesis. In: "The last great Ice Sheets" (GH Denton and T Hughes Eds.), Wiley, New York, 319-369 (1981).

[38] Whitehouse, I. E., Chinn, T. J. & Hoefle, H. C. Radiocarbon dates from raised beaches, Terra Nova Bay, Antarctica. *Geologisches Jahrbuch* **E38**, 321-334 (1989).

[39] Lorenzini, S., Olmastroni, S., Pezzo, F., Salvatore, M.C. & Baroni, C. Holocene Adélie penguin diet in Victoria Land, Antarctica. *Polar Biol.* **32**, 1077-1086 (2009).

[40] de Bruyn, M. *et al.* Rapid response of a marine mammal species to Holocene climate and habitat change. *PLoS genet.* **5**, doi: 10.1371/journal.pgen.1000554 (2010).

[41] de Bruyn, M. *et al.* Rapid increase in southern elephant seal genetic diversity after a founder event. *Proc. R. Soc. B.* **281**, doi:10.1098/rspb.2013.3078 (2014).

[42] Nichols, R. L. Coastal geomorphology, McMurdo Sound, Antarctica. *J. Glaciol.* **7**, 449-478 (1968).

[43] Hall, B. L., Baroni, C. & Denton, G. H. Holocene relative sea-level history of the southern Victoria Land coast, Antarctica. *Glob. Plan. Chang.* **42**, 241-263 (2004).
